# Kienböck's Disease in a Patient With Cerebral Palsy: Case Report and Literature Review

**DOI:** 10.7759/cureus.88286

**Published:** 2025-07-19

**Authors:** Paula Sousa, Mafalda Reis, Andreia Ferreira, Domingues Rodrigues, Mafalda Santos

**Affiliations:** 1 Orthopaedics and Traumatology, Unidade Local de Saúde de Vila Nova de Gaia/Espinho, Vila Nova de Gaia, PRT

**Keywords:** avascular necrosis, cerebral palsy, kienböck's disease, lunate, pain

## Abstract

Kienböck's disease is a rare condition characterized by avascular necrosis of the lunate bone, with a multifactorial etiology that remains not fully understood. Since its first reported association with cerebral palsy (CP), several factors have been suggested to contribute to its higher prevalence in this population. We present the case of a 17-year-old adolescent with CP and spastic-dyskinetic tetraparesis who developed left wrist pain without any history of trauma. Initial radiographs raised suspicion of lunate mild sclerosis, and magnetic resonance imaging confirmed the diagnosis of Kienböck's disease. The patient was treated with botulinum toxin injections, resulting in pain relief and functional improvement. A review of the literature supports the observation that Kienböck's disease may be more prevalent in individuals with CP than in the general population, although it is likely underdiagnosed. Conservative management, including the use of botulinum toxin A (BoNT-A), may be effective in controlling pain and preserving function. Early recognition of Kienböck's disease in this population is essential for appropriate and individualized management, taking into account each individual’s functional and cognitive profile.

## Introduction

Kienböck's disease is a disorder of the lunate bone caused by vascular compromise, resulting in avascular necrosis [1]. It was first described in 1910 by Austrian radiologist Robert Kienböck, who identified osteomalacia of the lunate [2]. Cerebral palsy (CP), which affects approximately 2-3 per 1,000 live births, has multiple etiologies that impact normal brain function to varying degrees. Movement disorders are the most common manifestations in CP and are typically classified as spasticity, dyskinesia, ataxia, or mixed/other types [3]. Characteristic upper limb deformities in CP include wrist flexion, often with ulnar deviation, due to the dominance of spastic ulnar deviators over their radial counterparts [4]. Since the first case series reporting Kienböck's disease in CP patients in 1977 [5], the association between these two conditions has been increasingly documented in case reports and series. In CP, Kienböck's disease commonly presents alongside spastic palmar wrist flexion [6]. The increased muscle tone in these patients subjects the radiocarpal joint to higher pressures than in healthy individuals [7], which may compromise lunate vascularization by compressing its blood supply [8]. Additionally, pain is frequently reported by individuals with CP, with upper limb pain accounting for about 25% of cases and often linked to overuse [6]. This can obscure or delay the diagnosis of Kienböck's disease.

The objective of this study is to describe a clinical case of Kienböck's disease in an adolescent with CP and to review the existing literature on the condition in this population. The review includes discussion of epidemiology, etiology, clinical presentation, diagnostic methods, classification, and treatment options.

## Case presentation

We present the case of a 17-year-old adolescent with CP, characterized by spastic tetraparesis with an associated dyskinetic component. In 2021, the patient presented to the emergency department (ED) with a complaint of worsening left wrist pain over the previous weeks, which had become progressively constant. Neither the patient nor the caregiver reported any history of trauma to the wrist. Prior to the onset of symptoms, the patient had symmetrical wrist mobility, with full flexion and extension limited to 45°. On physical examination in the ED, both wrists were in a flexed resting posture, with mild edema noted on the left side. The patient exhibited tenderness to palpation and experienced pain with both active and passive wrist movements. Range of motion was reduced on the left side compared to the right, with extension limited to 20°.

**Figure 1:**
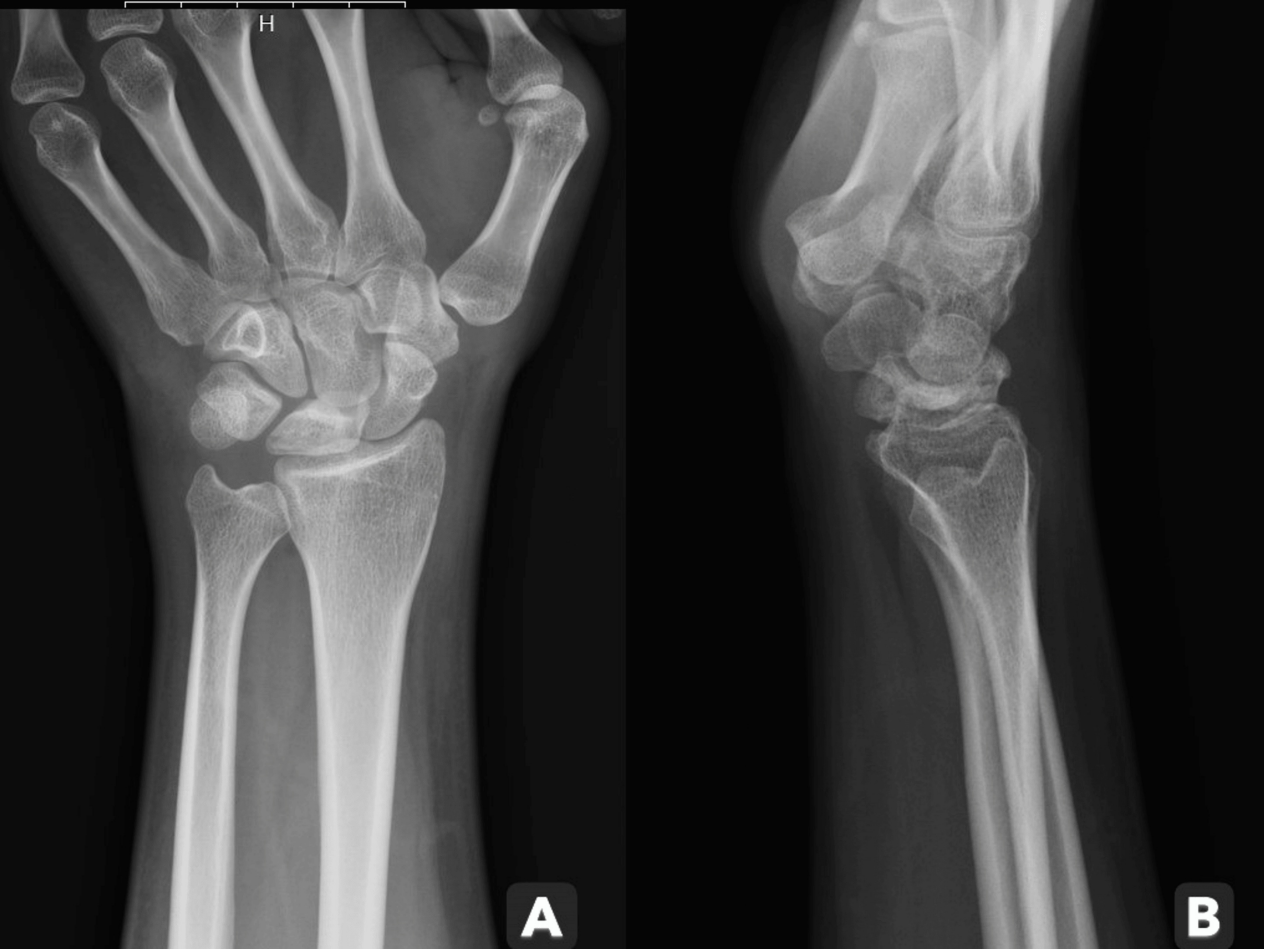
Initial left wrist radiograph indicating lunate mild sclerosis. (A) Anteroposterior view. (B) Lateral view.

An initial wrist radiograph raised suspicion of mild lunate sclerosis (Figures 1A, 1B). The patient was discharged with a recommendation for clinic follow-up, along with instructions for pain management, rest, and elevation.

**Figure 2:**
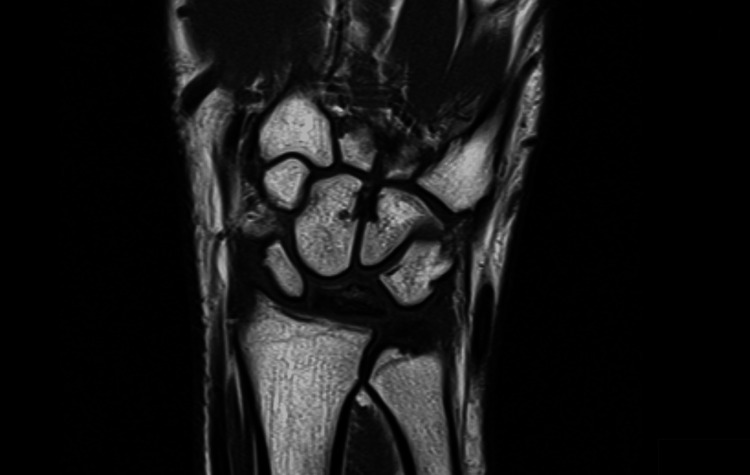
MRI of the left wrist. Coronal T1-weighted MR image shows hypointensity of the lunate bone, confirming the diagnosis of Kienböck's disease.

Due to persistent wrist pain and swelling after two additional weeks of conservative treatment, along with tenderness over the lunate on physical examination during follow-up, a magnetic resonance imaging (MRI) was requested. The MRI revealed hypointensity of the lunate bone on T1-weighted images (Figure 2), a finding consistent with avascular necrosis, confirming the diagnosis of Kienböck's disease. Based on the presence of sclerosis already noted on the X-ray, the condition was classified as stage II according to the Lichtman classification [9].

**Figure 3:**
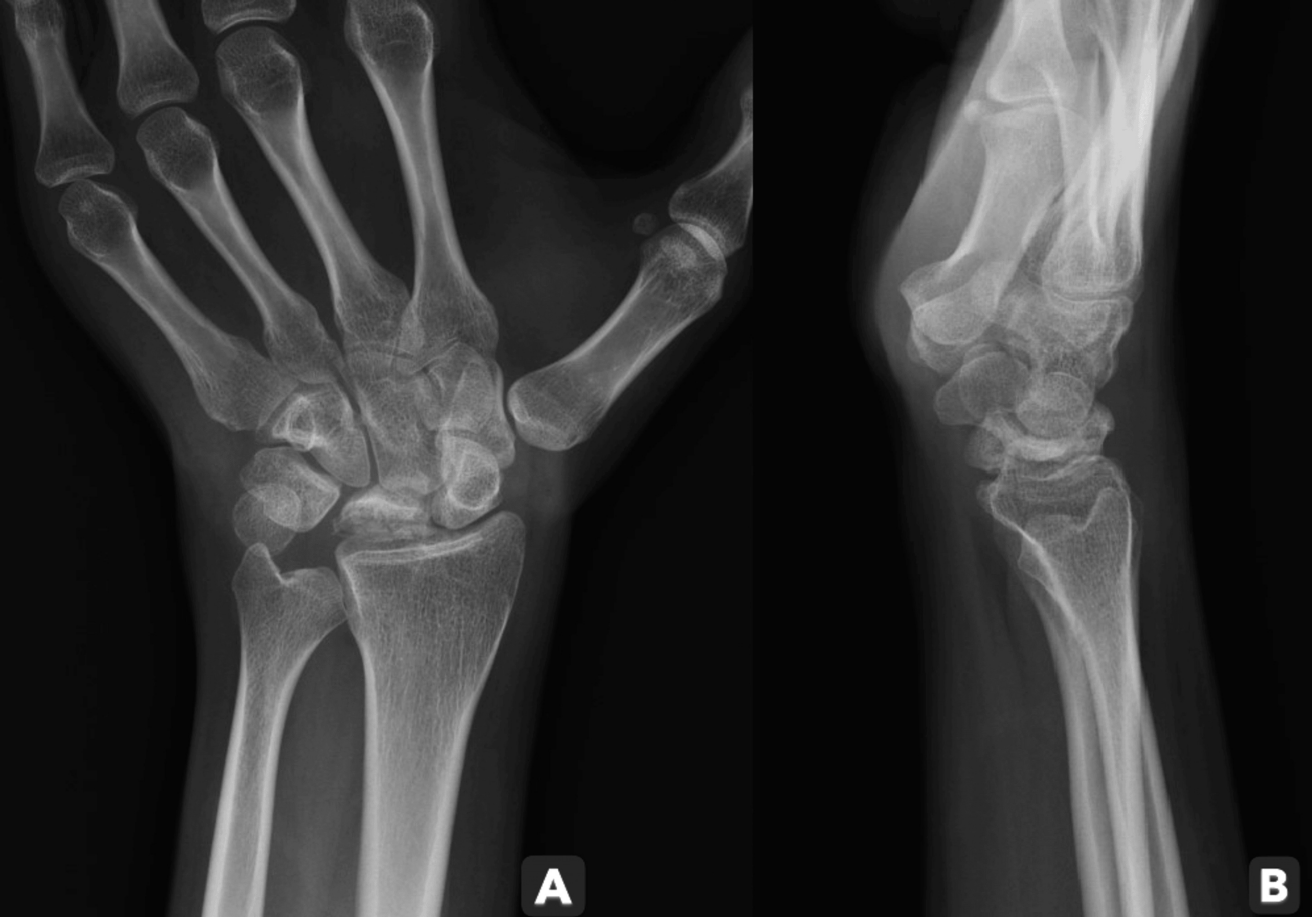
Left wrist radiographs at three-year follow-up with progression of Kienböck's disease. (A) Anteroposterior view. (B) Lateral view.

The patient was treated with botulinum toxin A (BoNT-A) injections into the flexor carpi ulnaris, flexor carpi radialis, and flexor digitorum superficialis, along with orthotic support and physical rehabilitation. This resulted in gradual pain relief and functional improvement. At the most recent clinic evaluation, three years after treatment, the patient had pain-free, symmetrical wrist mobility. Although radiographs showed progression of Kienböck's disease (Figures 3A, 3B), he remained asymptomatic in daily activities. The patient and his caregiver declined further surgical treatment due to concerns about possible functional decline.

## Discussion

Epidemiology

The higher incidence of Kienböck's disease in patients with CP compared to the general population was first described by Rooker and Goodfellow in 1977 [5], who reported a prevalence of 9.4% among 53 residents of a care institution for individuals with CP [5]. Subsequent studies have shown radiographic prevalence rates ranging from 2.7% to 8.3% in similar populations [7,10]. In contrast, the prevalence in the general population is reported to be between 0.006% and 0.5%, with a higher frequency in males aged 20 to 40 years [11,12]. Among the reported cases in the literature where age at diagnosis was available, the mean age was 30.3 years, with 12 out of 20 cases (60%) diagnosed between 20 and 40 years old [5-8,13-16] (Table 1).

**Table 1:** Case reports and case series in the literature of Kienböck's disease in patients with cerebral palsy. Case reports and case series including data on patient characteristics, Lichtman classification at diagnosis, and treatment approaches. M: male, F: female, CP: cerebral palsy, D: dyskinetic, S: spastic, Mx: mixed, NR: not reported.

Author (year)	Sex	Age	CP type	Ulnar variance	Lichtman classification	Treatment
Rooker and Goodfellow (1977) [5]	M	17	D	Minus	IV	NR
	M	28	D	Zero	II	NR
	F	17	S	Minus	III	NR
	F	26	Mx	Minus	III	NR
	F	20	S	Minus	III	NR
Gallien et al. (2010) [6]	M	28	D	Minus	II	Injection of botulinum toxin into the flexor carpi radialis
Joji et al. (1993) [7]	F	13	Mx	Minus	I	NR
	M	24	Mx	Minus	II	NR
	F	32	Mx	Zero	IIIB	NR
	F	36	Mx	Minus	IV	NR
	M	36	Mx	Minus	IIIB	NR
De Smet (2001) [8]	F	48	D	Zero	III	Proximal row carpectomy
Greene (1997) [13]	F	11	NR	Minus	NR	NR
Asami et al. (1992) [14]	M	58	S	Plus	IIIB	NR
	M	55	S	Minus	IV	NR
	M	35	S	Minus	I	NR
	NR	43	S	Zero	I	NR
	NR	33	S	Zero	I	NR
Leclerq and Xarchas (1998) [15]	F	25	S	Minus	IIIB	Proximal row carpectomy
Senda et al. (2010) [16]	M	20	D	Zero	IIIB	Closing wedge osteotomy of the radius with volar plating

Etiology

Kienböck's disease is believed to have a multifactorial etiology, with no clear consensus on its exact cause [11]. Factors implicated in its pathophysiology include fractures, repetitive minor trauma, negative ulnar variance, decreased radial inclination, lunate bone geometry, and conditions that compromise its vascular supply [7,11]. Several hypotheses have been proposed to explain the higher prevalence of Kienböck's disease in individuals with CP. These include a higher frequency of negative ulnar variance [10], reduced vascularization of the lunate due to fixed wrist flexion [5], repetitive microtrauma related to spasticity [7,17], and increased dynamic compression forces on the lunate, between the capitate and radius, resulting from elevated muscle tone [14]. In a radiographic study, Nishioka et al. reported negative ulnar variance in 18.2% of CP patients, compared to just 1.2% in the general Japanese population [18]. Among the 20 reported cases in the literature, 13 exhibited negative ulnar variance (65%), 6 had neutral variance, and only 1 had positive variance. As for CP types, 8 patients had predominantly spastic features (40%), 6 were classified as mixed (30%), 5 were dyskinetic (25%), and 1 case did not specify the type [5-8,13-16] (Table 1).

Clinical presentation

The symptoms of Kienböck's disease in patients with CP are generally similar to those seen in the general population, including dorsal wrist pain triggered by movement and palpation, swelling, decreased range of motion, and reduced grip strength. As the condition advances, pain may subside while joint stiffness becomes more prominent [12]. However, in individuals with CP, pain is already a common complaint, and upper limb discomfort may also stem from spasticity alone, often exacerbated by the use of crutches or wheelchairs [3,6]. This overlap can contribute to the underrecognition and underdiagnosis of Kienböck's disease in this population. Therefore, in cases of worsening wrist pain accompanied by swelling and functional decline, especially in the absence of trauma, Kienböck's disease should be considered as part of the differential diagnosis.

Diagnosis and classification

Diagnosis is primarily imaging-based, relying on radiographic findings, with CT or MRI providing additional information when needed [12]. Radiographs are useful for evaluating changes in bone density, lunate collapse or fragmentation, and degenerative changes in adjacent carpal bones, key features used in staging the disease using Lichtman’s classification [19]. CT scans can detect early signs such as subtle fracture lines, sclerosis, and early flattening of the radial inclination. MRI serves as an important complementary tool, capable of identifying early bone signal changes even when radiographs appear normal. A hypointense signal on T1-weighted images is considered an early and specific indicator of Kienböck's disease, while a T2 hypointense signal typically indicates disease progression [12]. In many cases, imaging with radiographs and/or MRI is only pursued when symptoms worsen, particularly with increasing pain, wrist swelling, and reduced function.

Treatment

In general, conservative treatment is recommended for mild cases of Kienböck’s disease (Lichtman stage I), with moderate efficacy. This typically involves rest, immobilization, and nonsteroidal anti-inflammatory drugs (NSAIDs) [20]. For more advanced cases, radial shortening osteotomy to correct negative ulnar variance is the most commonly performed surgical procedure and has shown good outcomes. Other surgical options for moderate cases (Lichtman stages II and III) include ulnar lengthening, vascularized bone grafting, lunate excision (with or without reconstruction), and various forms of carpal arthrodesis [19,20]. Salvage procedures, such as proximal row carpectomy and total wrist arthrodesis or arthroplasty, are reserved for severe or refractory cases (Lichtman stage IV) [20].

Among the reviewed literature, only four clinical cases reported treatment details in CP patients with Kienböck’s disease. In the mildest case, a 28-year-old patient with stage II disease was treated with BoNT-A injections into the flexor carpi radialis every three months, along with intermittent splinting. Pain relief occurred within 15 days, with no further need for analgesics over a two-year period, and no loss of strength or function was reported [6]. Another case involved a 20-year-old patient with stage IIIB disease who, after six months of conservative management, underwent a radial wedge osteotomy with volar plate fixation. Six years postoperatively, the patient reported significant pain improvement, despite persistent involuntary muscle contractions, and maintained stable function without radiographic progression [16]. In a third case, a 25-year-old patient with stage IIIB disease underwent proximal row carpectomy and synovectomy, followed by four weeks of immobilization. At 18 months, the patient showed improved function and pain control, with only occasional mild discomfort [15]. The final case involved a 48-year-old woman with stage III disease who received a proximal row carpectomy combined with flexor carpi ulnaris transfer into the extensor carpi radialis brevis. However, the clinical outcome was not described by the authors [8].

Flexion deformity of the wrist with ulnar deviation is common in CP and may be managed with non-surgical methods (e.g., occupational and physical therapy), spasticity treatment with BoNT-A, or surgery [3,4]. BoNT-A is widely used for spasticity management and may help relieve pain and delay surgical intervention. However, there is no strong evidence that it reduces contractures, decreases the need for surgery, or improves long-term function. For wrist spasticity, BoNT-A is typically injected into the flexor carpi ulnaris and flexor carpi radialis muscles. There are currently no established treatment guidelines for Kienböck’s disease in CP patients; treatment decisions should be based on symptom severity, disease stage, patient age, and, importantly, the individual’s functional and cognitive status [4].

## Conclusions

CP is now recognized as a risk factor for Kienböck’s disease. Although its prevalence is higher in CP patients compared to the general population, the number of reported cases remains low, likely due to underdiagnosis. Pain is the most common symptom reported by CP patients, and Kienböck’s disease should be considered when wrist pain occurs without a history of trauma, especially when accompanied by signs of inflammation. There are no specific diagnostic algorithms or standardized treatment protocols for Kienböck’s disease in CP, and management should always be tailored to the patient’s functional and cognitive status. BoNT-A has an important role in pain control for CP patients and may be a valuable tool in managing Kienböck’s disease. Due to concerns about functional decline, surgical treatment is often delayed in favor of conservative approaches focused on symptom relief.

In conclusion, our case report and literature review highlight the limited data available on Kienböck’s disease in CP, with most evidence derived from case reports or small series. Increased awareness and further research are needed to support timely diagnosis and guide appropriate treatment in this patient population.
